# Environmental Conditions Associated with Elevated *Vibrio parahaemolyticus* Concentrations in Great Bay Estuary, New Hampshire

**DOI:** 10.1371/journal.pone.0155018

**Published:** 2016-05-04

**Authors:** Erin A. Urquhart, Stephen H. Jones, Jong W. Yu, Brian M. Schuster, Ashley L. Marcinkiewicz, Cheryl A. Whistler, Vaughn S. Cooper

**Affiliations:** 1 Northeast Center for *Vibrio* Disease and Ecology, University of New Hampshire, Durham, New Hampshire, United States of America; 2 Department of Molecular, Cellular, and Biomedical Sciences, University of New Hampshire, Durham, New Hampshire, United States of America; 3 Department of Natural Resources and the Environment, University of New Hampshire, Durham, New Hampshire, United States of America; 4 Department of Microbiology and Molecular Genetics, University of Pittsburgh School of Medicine, Pittsburgh, Pennsylvania, United States of America; Argonne National Lab, UNITED STATES

## Abstract

Reports from state health departments and the Centers for Disease Control and Prevention indicate that the annual number of reported human vibriosis cases in New England has increased in the past decade. Concurrently, there has been a shift in both the spatial distribution and seasonal detection of *Vibrio* spp. throughout the region based on limited monitoring data. To determine environmental factors that may underlie these emerging conditions, this study focuses on a long-term database of *Vibrio parahaemolyticus* concentrations in oyster samples generated from data collected from the Great Bay Estuary, New Hampshire over a period of seven consecutive years. Oyster samples from two distinct sites were analyzed for *V*. *parahaemolyticus* abundance, noting significant relationships with various biotic and abiotic factors measured during the same period of study. We developed a predictive modeling tool capable of estimating the likelihood of *V*. *parahaemolyticus* presence in coastal New Hampshire oysters. Results show that the inclusion of chlorophyll *a* concentration to an empirical model otherwise employing only temperature and salinity variables, offers improved predictive capability for modeling the likelihood of *V*. *parahaemolyticus* in the Great Bay Estuary.

## Introduction

**Fig 1:**
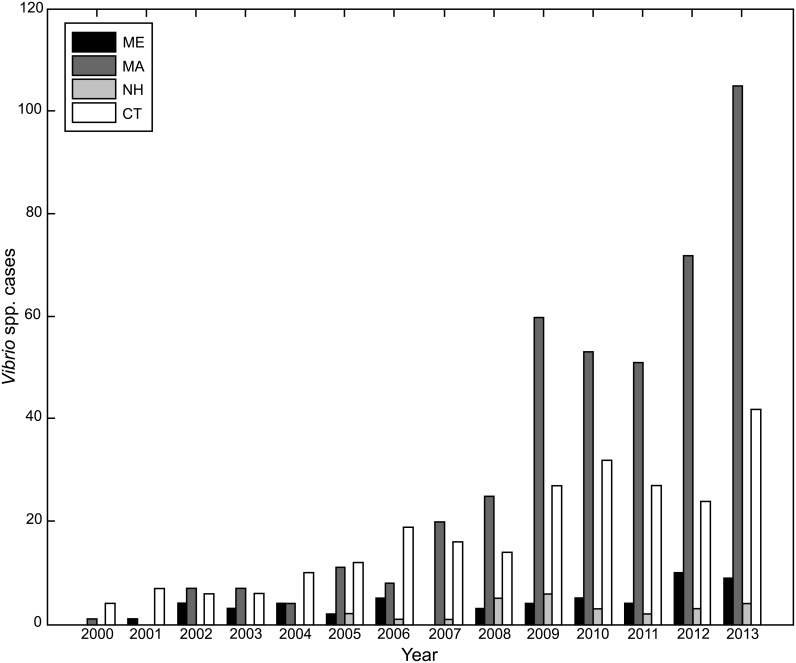
Annual cases of vibriosis in humans for Maine (ME), Massachusetts (MA), New Hampshire (NH), and Connecticut (CT) for 2000 through 2013. Zero cases are represented by missing vertical bars. Identified species include *V*. *parahaemolyticus*, *V*. *vulnificus*, *V*. *cholerae*, *V*. *alginolyticus*, *V*. *fluvialis*, and ‘unknown’.

Despite the cool coastal water temperatures characteristic of the New England states in Northeast United States, *V*. *parahaemolyticus* and other potentially pathogenic *Vibrio* spp. are recurrently detected in regional coastal ecosystems during the warm summer months, and essentially non-detectable during cold winter months [1–8]. *V*. *parahaemolyticus* is a free-living, halophilic, gram-negative bacterium found in coastal environments worldwide [9–11]. With an estimated 30,000 cases per year, *V*. *parahaemolyticus* is the most common bacterial source of seafood-borne gastrointestinal illness in the United States [12], and the incidence of *V*. *parahaemolyticus* cases nationwide is increasing [13]. The incidence of vibriosis in New England follows the seasonality of *Vibrio* spp. abundance in coastal waters, and species such as *V*. *parahaemolyticus* are regularly detected in shellfish harvested for consumption during warm months [14–18]. Though reported cases of human *Vibrio* infection are relatively infrequent, reports from state health departments [19–22] and the Centers for Disease Control and Prevention (CDC; [23]) indicate the annual number of reported human *Vibrio* infections in the New England region dramatically increased in the past decade (Fig 1), especially in Massachusetts and Connecticut where there were a combined 5 cases in 2000 compared to 147 cases in 2013. Several outbreaks of *V*. *parahaemolyticus* illnesses were associated with warmer than usual regional ocean temperatures and the regional invasion of ST36-O4:K12 strains [23–25]. The increase in the frequency of outbreaks and reported infections has caused harvest closures and economic losses to the shellfish industry, and led all New England states to initiate *V*. *parahaemolyticus* management plans [26]. Robust forecasting of *Vibrio* presence, concentrations and associated environmental conditions would greatly aid the management of shellfish harvesting for both preventing disease and maintaining a healthy shellfish industry.

The relationship between total abundance of *V*. *parahaemolyticus* and risk of disease to shellfish consumers has been documented [12], yet it remains an evolving question given recent insights into the nature of pathogenic strain emergence [24,25,27]. There are several reference *V*. *parahaemolyticus* concentrations that imply risk thresholds, including the level to which post-harvest shellfish processing approaches need to reach to be considered effective [30 MPN g^-1^; [28]], and the Health Canada limit for minimal risk conditions [100 MPN g^-1^; [29]]. Thus, public health risk concerns remain tied to *V*. *parahaemolyticus* concentrations as a basis for management approaches to protect public health, although actual risk is associated not only with pathogenic strains, but also, dose and immune condition of the consumer, and hence the relationship of risk to total *V*. *parahaemolyticus* concentrations has limitations [1]. *V*. *parahaemolyticus* strains containing virulence-associated markers are also rare in the environment, and this is especially true for the Great Bay estuary [25], making it necessary to base models on total *V*. *parahaemolyticus* detection.

In New England, where *Vibrio* spp. are an increasing public health concern, several studies [1,3–5,8] have documented the relationship between bacterial levels and environmental conditions. Whereas the environmental range of conditions that dictate *V*. *parahaemolyticus* abundance can differ by study region [30], they generally increase in abundance when water temperatures exceed 15°C and with salinity between 5 and 25 ppt [5,15]. These factors, and temperature in particular, also influence the pathogenic potential of different *V*. *parahaemolyticus* strains [31]. Other environmental measures have also been associated with variations in *V*. *parahaemolyticus* levels, including turbidity, suspended sediments, nutrients, and dissolved organic carbon [5,32,33]. Furthermore, *V*. *parahaemolyticus* populations are influenced by estuarine microbial communities, particularly in association with plankton where chitin and organic exudates are enriched and available for stimulating rapidly growing *V*. *parahaemolyticus* populations [34–37].

The main research objectives of this present study are to first advance the understanding of the associations between *V*. *parahaemolyticus* and environmental parameters, and second, to develop algorithms capable of predicting the likelihood of *V*. *parahaemolyticus* presence based on those associations in the Great Bay Estuary. Here we present empirical algorithms for estimating the probability of *V*. *parahaemolyticus* presence in the Great Bay Estuary, NH. This study builds upon existing environmentally-based pathogen prediction models [14,18,38–41] by incorporating not only abiotic but also biotic-related predictors to estimate *V*. *parahaemolyticus* presence in the coastal waters of the Northeast United States. Successful development of a *V*. *parahaemolyticus* likelihood algorithm will enable future development of more detailed and quantitative *Vibrio* spp. models capable of providing useful information for managers, researchers, and public health practitioners in the Gulf of Maine, New England and beyond.

## Materials and Methods

### Study Area

**Fig 2:**
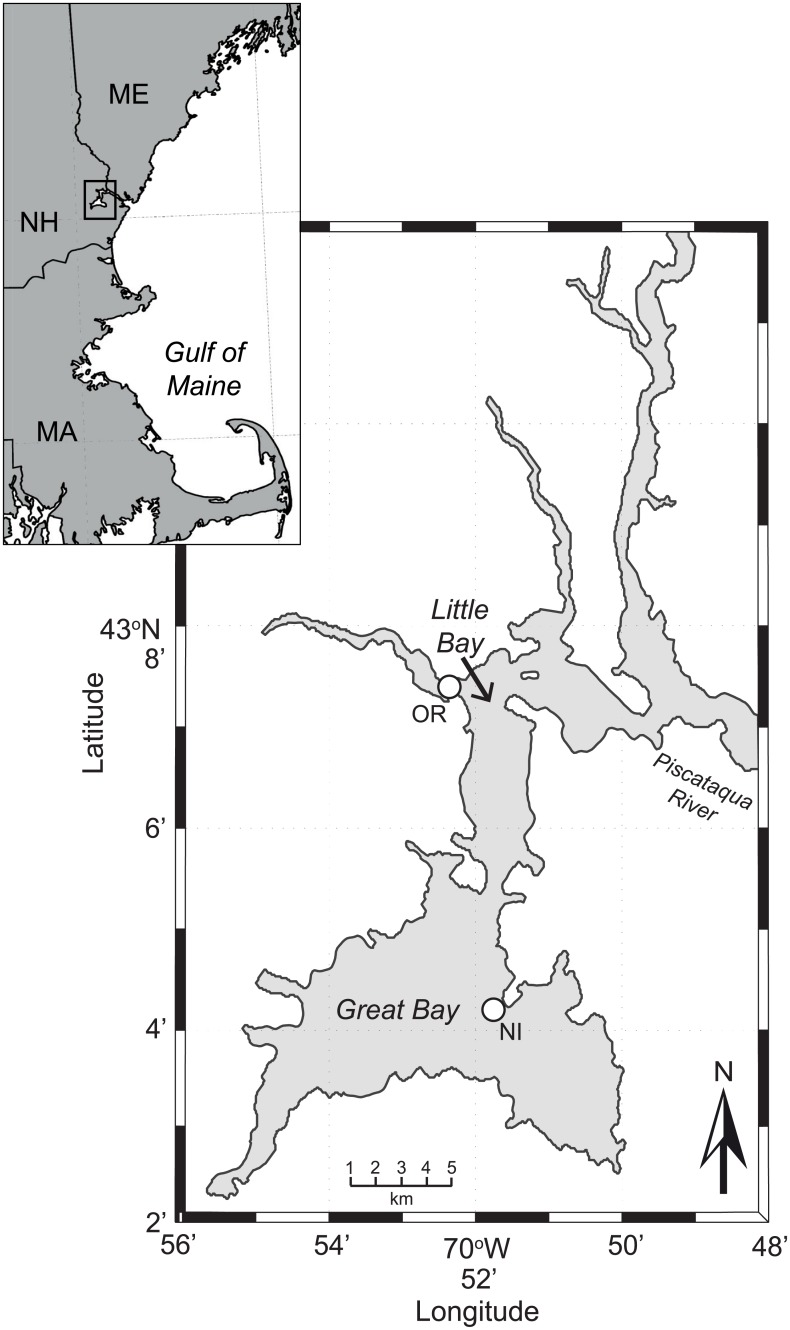
Map of Great Bay Estuary: white circles represent the sampling stations for this study.

Located within the Gulf of Maine watershed, the Great Bay Estuary (GBE) extends inland from the mouth of the Piscataqua River near Kittery, ME through Little Bay and eventually into Great Bay (~25 km; Fig 2). The GBE has deep, narrow channels with strong tidal currents, and wide, shallow mudflats. The physical transport regime of the Great Bay Estuary follows the classical estuarine circulation model for drowned river valley estuaries. Five tributaries, the Lamprey, Squamscott, Oyster, Bellamy, and Winnicut rivers account for the freshwater inflow into the Great Bay and Little Bay. Water surface temperatures range from local wintertime lows of <0°C to summertime highs of >29°C. Furthermore, salinity varies both seasonally and spatially throughout the estuary with values ranging from 0 to 28 ppt. The two study sites, Oyster River and Nannie Island, represent tributary and open bay conditions, respectively, and have been shown to vary in *Vibrio* spp. presence and abundance [5].

### *in situ* Collection and Sampling Procedures

American oysters (*Crassostrea virginica*) were obtained from 2007 through 2013 from two study sites in the Great Bay Estuary (Fig 2). Pooled samples of 12 live wild oysters were collected bi-weekly during warmer season (June-September) and monthly during the colder season (October-May). Oysters, collected using oyster tongs from boats over a one hour time period within 0.5 h of low tide at each site, were immediately stored in coolers with ice packs on-board for transport to the laboratory. All samples were collected within 45 minutes of low tide to ensure consistency of sampling conditions between sites on each sample data and between sample dates. A total of 143 *in situ* samples were collected for bacteriological analysis that was carried out within 2 to 4 hours at the University of New Hampshire.

Environmental data used in the statistical analyses were collected as part of the Great Bay National Estuarine Research Reserve (GBNERR) System Wide Monitoring Program (SWMP). Temperature (Temp), salinity (Saln), dissolved oxygen (DO), pH, and turbidity (Turb) were collected by datasondes deployed in the Great Bay from April-December with 15 minute readings of an array of environmental parameters (Table 1). Precipitation data (Rain) was acquired from several weather stations (http://cdmo.baruch.sc.edu/get/export.cfm; http://www.weather.unh.edu) in the Great Bay region. Surface (0.5 m depth) water temperature, salinity, dissolved oxygen, and pH data were also measured using YSI 6600 and EXO data sondes (YSI Inc., Yellow Springs, Ohio) at the time of sampling to check the accuracy of datasonde readings. In addition, analysis included monthly nutrient data, including chlorophyll *a* (Chla), phosphate (PO_4_), and total dissolved nitrogen (TDN), were collected by the GBNERR SWMP (http://cdmo.baruch.sc.edu/get/export.cfm). Chlorophyll *a* was determined using EPA Method 445.0 [42]. No specific permissions were required for retrieval of environmental data at either location as the GBNERR SWMP provides free publicly available data. The fieldwork performed in this study did not involve endangered or protected species.

**Table 1:** Correlation coefficients for log-transformed *V*. *parahaemolyticus* abundance and selected environmental variables.

	Temp	Saln	DO	pH	Turb	TDN	Chla	PO^4^	Rain
*R*	0.49	0.27	0.38	0.12	0.13	0.14	0.29	0.12	0.00
*p-value*	2.3E^-09^	1.8E^-03^	6.1E^-06^	0.17	0.14	0.10	8.6E^-04^	0.18	0.98

Abbreviations correspond to environmental parameter names in rows. Significant correlations are at the alpha (p<0.05) level.

### *V*. *parahaemolyticus* Isolation and Identification

After the outer shells were scrubbed and cleaned, and the oysters shucked aseptically with a sterilized oyster knife, pooled tissue from 12 oysters was processed for enumeration of *V*. *parahaemolyticus* via a 3-tube MPN enrichment method following the FDA Bacteriological Analytical Manual (BAM; [43]), coupled with culture-based and polymerase chain reaction (PCR) methods used to confirm the presence of *V*. *parahaemolyticus* [44]. The oysters were added to a sterile beaker (liquor and meat), weighed and diluted with buffered peptone water (BPW: 10 g peptone, 5 g NaCl, 3.5 g Na_2_HPO_4_, 1.5 g KH_2_PO_4_ per L) and homogenized for 30 seconds on low speed and 60 seconds on high speed. From 2007–2010, ten grams of homogenate was added to three tubes containing 10 mL of alkaline peptone water (APW, pH 8.6, 1% NaCl), and 1 gram of homogenate was added into each of three separate APW tubes and into a separate dilution tube containing 9 mL of BPW. From 2011–2013, ten grams of homogenate was added to three tubes containing 10 mL of APW as the first dilution step. A serial 10-fold dilution series in BPW tubes down to 10^−6^ was used to ensure detection of high concentrations during warm months. One mL aliquots of diluted homogenate were added to 9 mL of APW in three tubes at each dilution and all tubes were incubated at 37°C overnight (18–20 hours).

APW tubes that were turbid after incubation were scored as positive for growth and streaked onto selective agar. From 2007–2010, cultures were quadrant streaked to TCBS agar (BD, Franklin Lakes, NJ), whereas from 2011–2013, APW positive tubes were quadrant streaked onto CHROMagar Vibrio (CHROMagar, Paris, France); both media were incubated at 37°C for 18–20 hours. Putative *V*. *parahaemolyticus* isolates (Sucrose negative colonies-TCBS; purple colonies-CHROMagar Vibrio) were further isolated onto tryptic soy agar (TSA; BD) and incubated at room temperature for 18–20 hours.

Isolates from the APW enrichment tubes that were putatively identified as *V*. *parahaemolyticus* based on colony color were subsequently subjected to species identification by a standard PCR-based assay using the species-specific gene (*tlh*) to determine *V*. *parahaemolyticus* MPN values [44]. The putative isolates from TCBS or CHROMagar Vibrio, were re-streaked onto TSA agar. Colonies were suspended in 1 mL Molecular Biology Grade Water (Phenix Research Products), then boiled at 100°C for 10 minutes and centrifuged for 5 minutes at 8000rpm [44]. A 2μL sample of the supernatant was added to 13 μL Mastermix in 0.2mL PCR tube. The Mastermix was composed of 1X iQSupermix (Bio-Rad, Hercules, CA) containing dNTPs, 25 U/ml iTaq DNA polymerase, 3 mM MgCl_2_ and then 125 nM of species-specific *tlh* primers [44] and nuclease free water to a total volume of 25 μL. For efficiency, some PCR assays were scaled down to 10 μL. The PCR conditions were conducted as described previously [44]. The presence of the correct size amplicon (*tlh*) was determined by electrophoretic separation on 1.2% agarose gel with addition of Gel Red (Phenix Research Products, Candler, NC) under UV light and comparison to a standard strain (F11-3A) to confirm the presence of *V*. *parahaemolyticus* in any given MPN tube. MPN concentrations for each sample were then determined based on the presence of confirmed *V*. *parahaemolyticus* in MPN tubes based on the FDA BAM [43]. The limit of detection (LOD) was 0.018 MPN g^-l^.

### Statistical Methods

Datasonde water quality data from the 12 hours prior to oyster sampling were averaged to provide tide stage integrated data reflecting conditions that could affect *V*. *parahaemolyticus* concentrations, and to ensure dataset consistency for empirical model development. For purposes of temporal binning and ecological lag times, mean cumulative surface temperature and salinity observations were calculated in 12-hour (~1 tidal cycle) time increments.

Correlations between *V*. *parahaemolyticus* concentrations and *in situ* environmental data were calculated for all months at all sampling locations. The measure of linear association between log-transformed *V*. *parahaemolyticus* concentrations and paired environmental parameter data were calculated by Pearson’s product-moment correlation (R; [45]), with significant relationships determined by the degrees of freedom (n = 125) at an alpha level of 0.05. For the purposes of data normality, all MPN g^-1^ values for *V*. *parahaemolyticus* concentrations were log_10_ transformed prior to statistical analysis.

For the purposes of model fit and observation of data trends within the normal range of *V*. *parahaemolyticus* concentrations, outlying, relatively high *V*. *parahaemolyticus* concentration data points (determined by the absolute value of 2 times the standard deviation) were excluded from the following correlation analysis and model development and validation. Additional data points in which concurrent environmental data were not available were also excluded from the correlation analysis as well as model development and validation.

#### *V*. *parahaemolyticus* probability statistics

Generalized Linear Models (GLM; [46]) were used to determine the association of various biotic and abiotic factors with characteristics of *V*. *parahaemolyticus* distribution, namely probability of presence in the Great Bay Estuary. Following the methods of Urquhart et al. (2015) and safe post-harvest safety levels [28], observational bacteria data in oysters were transformed to binary presence/absence: cell count > 30 MPN g^-1^ ≡ presence, cell count < or = 30 MPN g^-1^ ≡ absence. Stepwise regression based on Akaike’s Information Criterion (AIC; [47]) was used to select the best-fit likelihood model, in which each explanatory variable was entered sequentially into the model, and selected variables were retained only if significant. The GLM algorithms were applied in the logistic (“logit”) form and statistical analysis was performed using the *stats* (version 2.14.0) R package [46] carried out in the R Statistical Software, version 3.1.2 [48]. For correlation analysis and model evaluation, significance was set at an alpha level of 0.05. To assess model predictive skill, the resulting top two models of the stepwise selection were tested in an out-of-bag [49] cross validation analysis. To ensure correct binary classification, a 0.5 prediction point was used. Final model selection was based on the optimization of five model assessment error indices: true positive rate (sensitivity as a percent; TPR), true negative rate (specificity as a percent; TNR), positive predictive value (PPV), negative predictive value (NPV), and Matthews Correlation Coefficient (MCC; [50]).

## Results

### Detection and Trends

**Fig 3:**
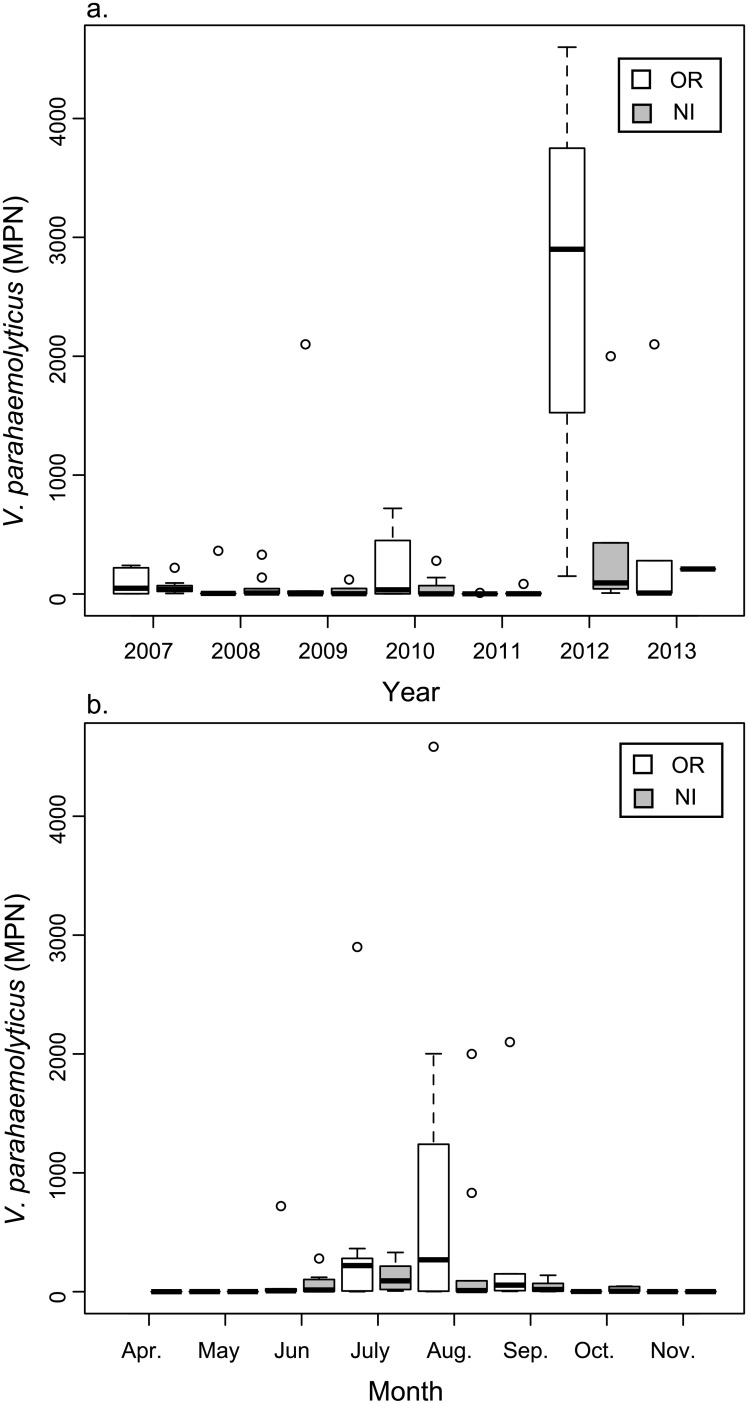
Boxplot showing *V*. *parahaemolyticus* concentration (MPN g^-1^) for station and year (A), and for station and month (B) for Oyster River (OR) and Nannie Island (NI). Hollow circles represent outliers; dashed vertical lines illustrate ICQ range; bold horizontal bars represent median *Vp* concentration value.

*V*. *parahaemolyticus* was detected seasonally in oysters each year at both sites (Fig 3A) and there was no effect of sampling location on *V*. *parahaemolyticus* concentration (p = 0.139). *V*. *parahaemolyticus* was detected (= > 0.018 MPN g^-1^) in 91 of 140 (65%) oyster samples, with concentrations ranging from 0.04 MPN g^-1^ to 4,600 MPN g^-1^. Detection during cold-water months (October through May) was 55% whereas warm-water month (June through September) detection was 72%. For most years, higher concentrations of bacteria in the GBE were first detected beginning in late May to June, after water temperatures rose to above ~15°C (Fig 3B). In oyster samples with *V*. *parahaemolyticus* detected, the median and geometric mean counts were 7.3 MPN g^-1^ and 12.3 MPN g^-1^, respectively. Thirty five (35%) of the samples exceeded 30 MPN g^-1^, a level below which oysters subject to post-harvest processing are considered safe [28]. As we expected, the levels of total *V*. *parahaemolyticus* bacteria detected in NH oysters are slightly lower, yet comparable to densities (10^3^−10^4^ MPN g^-1^) found in shellfish from other coastal regions [16,32,51–53]. Seven outlying data points and additional eight data points in which concurrent environmental data were not available were excluded from correlation analysis as well as model development and validation.

### Influence of Environmental Factors

Higher mean water temperature over the 60 hours prior to sampling was correlated with increased *V*. *parahaemolyticus* detection in oyster samples collected during all months (April through December). The median temperature when bacteria were detected was 19.7°C, compared to 17.2°C when bacteria were not detected. The seasonal trends in the onset of bacterial presence are similar to occurrences found Rhode Island [8] and the Chesapeake Bay [18,38,39], where *V*. *parahaemolyticus* densities increased in oysters following sustained high water temperatures. For salinity, an overall increase in mean values over the 72 hours prior to sampling was also linked to higher bacteria presence in oysters (24.4 ppt when present, 20.5 ppt when absent).

Log-transformed *V*. *parahaemolyticus* concentrations (MPN g^-1^) significantly correlated with mean water temperature (°C) over the previous 60 hours, mean salinity (ppt) over the previous 72 hours, mean dissolved oxygen over the previous 12 hours (mg/L), total dissolved nitrogen (mg/L), and chlorophyll *a* (μg/L) during all months (Table 1). Although the noted associations were significant (p<0.05), all of the correlation coefficients were relatively low (<0.49). Linear correlations between *V*. *parahaemolyticus* levels and mean turbidity (NTU) over the previous 12 hours, pH, phosphate (*PO*_*4*_; mg/L), and rainfall over the previous week (*Rain*; inches) were not statistically significant at a 0.05 alpha level (Table 1). Differences in environmental variables and their correlations with *V*. *parahaemolyticus* abundance between sampling sites were minimal.

### Predictive Model

Using stepwise regression for the probability of *V*. *parahaemolyticus* presence, based initially on minimum AIC values, the top two likelihood models were selected and compared for use in prediction and cross validation. In comparing statistical models, the AIC estimates the quality of each model (lower AIC value indicates higher quality), relative to each of the other models by assessing the trade-off between model complexity and goodness of fit [47]. The top-performing model (referred to as *Vp1*, (AIC) = 95.46) explains the probability of presence of *V*. *parahaemolyticus* in oysters and is composed of in situ temperature and salinity. The second best model, *Vp2*, (AIC) = 95.42 is composed of temperature, salinity, and chlorophyll *a*. Five evaluation indices were used to test each binary model’s suitability in out-of-sample prediction (Table 2). As model predictions are in the form of probabilities, we classified estimates as either present or absent based on a 0.5 prediction point. Albeit *Vp1* and *Vp2* are indistinguishable when comparing AIC values, *Vp2* provides greater predictive performance, with the MCC (0.46) for *V*. *parahaemolyticus* presence in out-of-bag model assessment. Furthermore, though predictions of the two models were not statistically different (p<0.05), *Vp2* outperformed *Vp1* across all evaluation indices, not including true negative rate in which the proportions for both models were equal (TNR = 0.91). These results indicated that the addition of chlorophyll *a* improves the model’s ability to correctly classify predicted *V*. *parahaemolyticus* observations, as well as helping to minimize Type 1 and Type 2 errors (see Table 2) for *V*. *parahaemolyticus* likelihood prediction.

**Table 2:** *V*. *parahaemolyticus* performance metrics.

Metric	*Vp1*	*Vp2*
AIC	95.46	95.42
MCC	0.33	0.46
TPR	0.37	0.52
TNR	0.91	0.91
PPV	0.56	0.64
NPV	0.83	0.86

AIC, Akaike’s Information Criterion; MCC, Matthews Correlation Coefficient; TPR, True Positive Rate; TNR, True Negative Rate; PPV, Positive Predictive Value; NPV, Negative Predictive Value.

**Fig 4:**
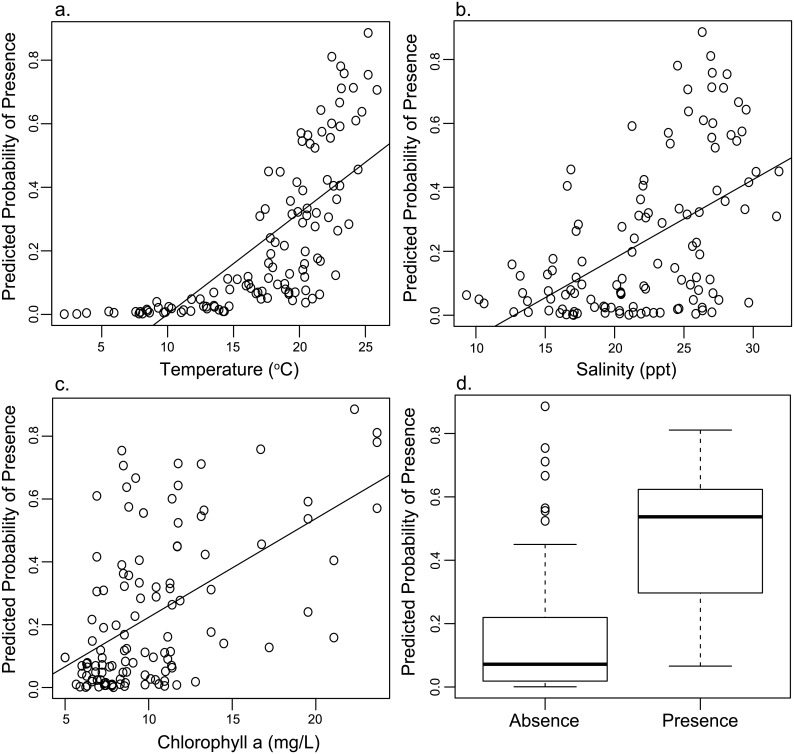
Plots showing the relationship between *Vp2* predicted *V*. *parahaemolyticus* probability and (A) surface temperature (°C), (B) salinity (ppt), and (C) chlorophyll *a* concentration (mg/l). Linear trend lines denoted by solid black line. Performance of *Vp2* binary classification presented as a boxplot comparing observed presence and absence with modeled probability (D).

To visualize the *Vp2* model response, we plotted *V*. *parahaemolyticus* likelihood against each significant model predictor (Fig 4). For mean surface water temperature for the 60 hours prior to sampling, a significant positive correlation (*R* = 0.73) was observed in the out-of-bag validation (Fig 4A). Likewise, we see a significant positive correlation (*R* = 0.52) between predicted *V*. *parahaemolyticus* likelihood and the mean salinity for the 72 h prior to sampling (Fig 4B). A positive correlation (*R* = 0.53) was found between chlorophyll *a* concentrations and predicted *V*. *parahaemolyticus* likelihood (Fig 4C). To assess the performance of binary classification, predicted probability of *V*. *parahaemolyticus* presence was split into observed bacteria presence (n = 89; mean probability = 0.45) and observed absence (n = 53; mean probability = 0.15 (Fig 4D)).

## Discussion

We present here an analysis of relationships between environmental parameters and *V*. *parahaemolyticus* presence and concentrations in oyster samples collected from two sites in the Great Bay Estuary over seven years.

Consistent with the results of previous studies from other locations, univariate correlation results demonstrate significant linear associations between sea surface temperature and *V*. *parahaemolyticus* densities in Great Bay oysters for all months [15–18,54]. In general, *V*. *parahaemolyticus* closely follows the seasonality of temperature in the estuary, with observed increases and decreases in concentrations as surface temperatures increased and decreased. Not surprisingly, prior to the removal of “cold-water months” observations (October through May), temperature is the dominant predictor of *V*. *parahaemolyticus* in the estuary, possibly masking out any associations with other environmental parameters. Furthermore, despite the correlation with temperature, many of the oyster samples harvested during summer months exhibit non-detectable or low *V*. *parahaemolyticus* levels, suggesting that while dominant, surface temperature may not be the only factor contributing to the abundance and frequency of *V*. *parahaemolyticus* in the Great Bay.

The relationship between salinity and *V*. *parahaemolyticus* abundance in the Great Bay also reflects the observations of previous studies [15,16,18,32,40,55–58] showing a significant association over a highly variable range of salinity samples. Due to the hydrodynamic nature of the Great Bay, oyster samples, and synchronous environmental and water quality measurements were taken during low tide to provide consistency in sample collection and to remove tidal stage as a variable at the two sites. Correlation analyses indicate that, consistent with many previous studies [16,59–62], although not all [55,63,64], chlorophyll *a* has a significant positive association with *Vibrio* spp. concentrations suggesting an association between plankton (related to chlorophyll *a* concentration) and *V*. *parahaemolyticus* in the estuary. Moreover, the addition of chlorophyll *a* to a *V*. *parahaemolyticus* likelihood model based previously only on temperature and salinity, improves the accuracy for prediction of *V*. *parahaemolyticus* likelihood in the Great Bay Estuary. Work currently in progress includes more research exploring the potential of zooplankton and phytoplankton to act as a reservoir for *Vibrio* spp. bacteria in the estuarine environment.

The empirical models presented here offer an updated platform for estimating likelihood of *V*. *parahaemolyticus* presence in the Great Bay estuary; however, we acknowledge several study limitations. First, the limited geographical area of the study restricted the *in situ* data to a fairly narrow range of high salinity values (mean: 22.3 ppt; std: 5.1), which also reflects the more saline conditions that are typical of warmer water conditions in the estuary. Due to the nature of the training data, extension of the model to less saline regions of the estuary, and/or to other coastal systems may require modification of the modeling approach, otherwise, the existing model could lead to increased prediction error. Secondly, for the purpose of model training and data display, outliers falling outside the interquartile range are excluded from the current model database. The dramatic intra-annual variability observed in *V*. *parahaemolyticus* concentrations in Great Bay oysters during the summer is also notable. Rapid growth rates and strain diversity, especially evident during warmer months [60], provides vibrios with a greater capacity to respond to favorable environmental conditions than during cold-water months.

We recognize that determining risk associated with consuming shellfish based on total *V*. *parahaemolyticus* concentrations may not be the best approach. Tracking tdh and/or trh genetic markers have been useful for estimating the presence of potentially pathogenic strains within total *Vp* populations, but these are not always associated with clinical cases [1,25]. These markers are also associated with a variety of *Vp* strains that may vary in virulence or environmental fitness. The best approach is to track pathogenic strains, and progress is being made in the Northeast United States in that regard to define regionally significant pathogenic strains [25,27]. As existing guidance levels for risk are based on total *V*. *parahaemolyticus* concentrations [28,29], our approach assists in determining when environmental conditions are favorable for *V*. *parahaemolyticus* presence. To explore the response and sensitivity of bacterial concentration to varying environmental conditions and geographic domains, work in progress includes employment and evaluation of region-specific empirical models for the prediction of *V*. *parahaemolyticus* abundance.

In summary, the current study examines the relationship between *V*. *parahaemolyticus* in oysters and various independent environmental parameters in the Great Bay Estuary. Strong predictive relationships were established to which we developed empirical models for the likelihood of *V*. *parahaemolyticus* presence. The work builds upon existing findings from other coastal regions by extending predictive modeling for *Vibrio* spp. bacteria into the Northeast United States. Results of this study confirm that inclusion of chlorophyll *a* concentration into a model otherwise employing only temperature and salinity, offers improved predictive capability for modeling the likelihood of *V*. *parahaemolyticus* in the Great Bay Estuary. Ongoing efforts to improve the understanding of region-specific conditions that can be used to inform risk models will be enhanced with similar small-scale modeling efforts in the Northeast that identify what may be area-specific environmental variables associated with *V*. *parahaemolyticus* presence and concentrations. Application of this approach to areas in New England where pathogenic *V*. *parahaemolyticus* strains are present and data of their detection [27] is generated will be useful to ongoing efforts by federal agencies to develop forecasting capacity.
